# The attack on universal health coverage in Europe: recession, austerity and unmet needs

**DOI:** 10.1093/eurpub/ckv040

**Published:** 2015-05-19

**Authors:** Aaron Reeves, Martin McKee, David Stuckler

**Affiliations:** 1 Department of Sociology, University of Oxford, Oxford, UK; 2 Department of Health Services Research and Policy, London School of Hygiene and Tropical Medicine, London, UK

More than 1.5 million extra people have unmet need for healthcare since the beginning of the economic crisis in Europe. The advent of the Great Recession has placed Europe’s health systems under severe pressure, with real terms cuts to funding in many countries.^1^ Accounts in the peer-reviewed literature and popular media have catalogued examples of vulnerable groups and individuals unable to access necessary care.^2^ Although there have been case-studies of Spain, Greece and other individual nations,^3^ to our knowledge there has been no systematic attempt to quantify changes in unmet need for medical care across the European Union. Here, using data from the EU-wide Statistics of Income and Living Conditions (EU-SILC), we quantify the increase in self-reported unmet need, a comparative measure of healthcare access defined as being unable to obtain care when people believed it to be medically necessary, in association with the Great Recession.^4^

From 2005 to 2008, prior to the Great Recession, the proportion of the EU population (a population-weighted average of individual national figures) reporting unmet need was falling, as shown in the figure 1. Between 2005 and 2008 self-reported unmet medical need fell by 2% points, from 5 to 3.1%. Then, coinciding with the onset of the recession in 2008, this trend reversed, to rise through the period of austerity in Europe, to reach 3.4% in 2012, the latest year for which data are available.

**Figure 1:**
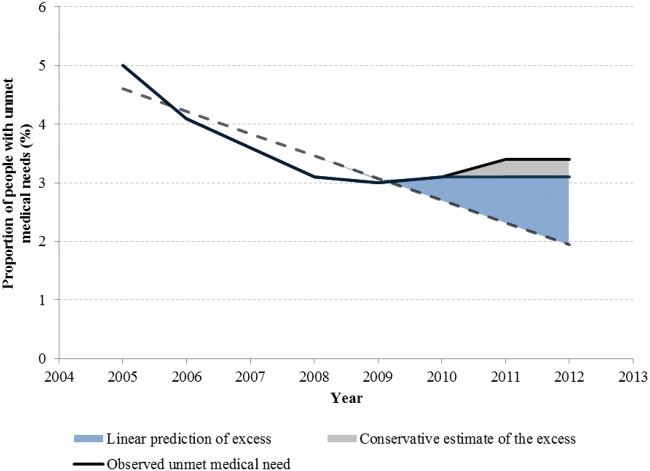
Observed and predicted unmet medical need across EU, 2005–12. Notes: Source: Eurostat

Using time-series discontinuity regression models, we then quantified the consequences of this reversal. In the years leading up to the Great Recession the prevalence of unmet medical need was falling at 0.4% points (95% CI: −0.16 to −0.58) each year (Table 1). After 2010, when austerity was implemented, unmet medical need began to rise in many countries, with an overall increase across the EU of 1.23% points (95% CI: 0.12 to 2.34) each year. These small percentage changes translate into a substantial population impact. In 2011, there were 502.5 million people in the EU. Taking the most conservative scenario, if we assume that the earlier decline in unmet need would simply have plateaued after 2010, then by 2011–12 the observed increase would represent an additional 1.5 million people who failed to obtain care they considered medically necessary. If, as is arguably more plausible, the previous decline had continued, then the difference would be even greater, totalling ∼7.3 million people.

This overall trend conceals national differences and not all countries experienced worsening access to healthcare. In Sweden, during the crisis period between 2007 and 2012, unmet medical need fell by 1.8% points, whereas in Belgium, in contrast, it rose by 1.7% points across this same period.

**Table 1:** Time trend analysis of 27 EU countries, 2005–12

Covariate	Proportion of people with unmet medical need (95% CI)
Annual trend, 2005–10	−0.37% points (−0.16 to −0.58)
Estimated excess unmet need during austerity, 2011–12	1.23% points (0.12 to 2.34)

Notes: Source: EuroStat.

Rising unmet needs also appeared concentrated in deprived groups. Again, taking the most conservative scenario, we found that an additional 100 000 people experienced unmet medical need in the highest income quintile (population-weighted average across each country) while an additional 600 000 people in the poorest groups were unable to access care they considered medically necessary, a 6-fold larger increase. A continuation of this trend would further widen inequalities in healthcare access in Europe.

The critical question for future research is what is reducing access to healthcare in some places but not in others? One hypothesis is that rising co-payments, which have expanded the cost of pharmaceuticals, outpatient care and A&E visits in some countries, are creating barriers to healthcare access in Europe. Despite clear evidence that such charges reduce both necessary and unnecessary utilization of care,^5^ they have been extended and expanded in the Czech Republic (2011), France (2010), Italy (2011), Latvia (2009), the Netherlands (2010) and Romania (2011), a process that has continued subsequently, as in Spain (2013). In contrast, nations that have reduced user fees, such as Croatia in 2011, have experienced declines in unmet medical need.^3^

Another hypothesis is that cost-saving reforms have reduced access to care. Cuts to healthcare services, involving closure of facilities (e.g. in Greece), reduced opening hours, and shrinking numbers of healthcare personnel can also worsen access to care.^6^^,^^7^ Several policy charges outside the health sector, such as reduced affordability of transport, might create further financial barriers to access.

In practice, it is likely to be a combination of factors, with the balance varying among countries. Thus, all of these factors combine in Greece, which may explain the marked increase in unmet medical need; between 2007 and 2011 unmet medical need attributed to greater costs of care rose by ∼39%, whereas unmet medical need attributed to transport costs was 2.8 times higher in 2011 than in 2007.

Finally, it is possible that recent policy reforms which restrict access of migrants and other vulnerable groups such as homeless persons and drug users, who tend to be under-represented in surveys, are taking a toll beyond what can be seen with EU-SILC data. In Spain, a royal decree in 2012 restricted access to the National Health Service for undocumented immigrants. The Czech Republic similarly has rewritten eligibility criteria to include only those who are permanent residents. In the UK, migrants from outside the European Economic Area will face additional costs to obtain healthcare services. By excluding certain groups from the social contract, states are undermining the principle of solidarity on which most European healthcare systems are based. Future targeted research is needed to investigate trends in unmet need among these high-risk groups.

Irrespective of which reasons are most important, our observation of rising unmet need is a cause for concern. Many European nations have long benefited from universal access to care. Even though Europe’s recessions may be coming to an end, this promise remains under threat.

## Funding

AR and DS are support by a Demetriq EU FP7 grant. This study was carried out with financial support from the Commission of the European Communities, grant agreement no. 278511. The study does not necessarily reflect the Commission’s views and in no way anticipates the Commission’s future policy in this area. DS is also funded by a Wellcome Trust Investigator Award.

*Conflicts of interest*: None declared.
